# Human Papillomavirus Prevalence and Herd Immunity after Introduction of Vaccination Program, Scotland, 2009–2013

**DOI:** 10.3201/eid2201.150736

**Published:** 2016-01

**Authors:** Ross L. Cameron, Kimberley Kavanagh, Jiafeng Pan, John Love, Kate Cuschieri, Chris Robertson, Syed Ahmed, Timothy Palmer, Kevin G.J. Pollock

**Affiliations:** Health Protection Scotland, Glasgow, Scotland, UK (R.L. Cameron, J. Love, S. Ahmed, K.G.J. Pollock);; University of Strathclyde, Glasgow (K. Kavanagh, J. Pan, C. Robertson);; Scottish Human Papillomavirus Reference Laboratory, Edinburgh, Scotland, UK (K. Cuschieri);; University of Edinburgh, Edinburgh (T. Palmer)

**Keywords:** human papillomavirus vaccine, prevalence, herd immunity, cervical cancer, cancer screening, viruses, vaccination, Scotland, HPV

## Abstract

In 2008, a national human papillomavirus (HPV) immunization program using a bivalent vaccine against HPV types 16 and 18 was implemented in Scotland along with a national surveillance program designed to determine the longitudinal effects of vaccination on HPV infection at the population level. Each year during 2009–2013, the surveillance program conducted HPV testing on a proportion of liquid-based cytology samples from women undergoing their first cervical screening test for precancerous cervical disease. By linking vaccination, cervical screening, and HPV testing data, over the study period we found a decline in HPV types 16 and 18, significant decreases in HPV types 31, 33, and 45 (suggesting cross-protection), and a nonsignificant increase in HPV 51. In addition, among nonvaccinated women, HPV types 16 and 18 infections were significantly lower in 2013 than in 2009. Our results preliminarily indicate herd immunity and sustained effectiveness of the bivalent vaccine on virologic outcomes at the population level.

## Abstract 2

Prevalence was reduced, and early evidence indicates herd immunity.

Human papillomavirus (HPV) vaccination programs are established in several countries worldwide (*1*–*3*). The national vaccination program in Scotland began in 2008; a bivalent vaccine that conferred protection against HPV types 16 and 18 was offered at school to girls 12–13 years of age (routine cohort). In addition, girls 13–17 years of age (3-year catch-up cohort) were offered the vaccine from September 2008 through August 2011. Starting in September 2012, the licensed quadrivalent vaccine replaced the bivalent vaccine in the program. Consequently, data in this article reflect the effects of the bivalent vaccine only. Since the 2008–09 school year, receipt of all 3 doses was >90% for girls in the routine cohort, and during 2008–2011, it was lower (65%) for girls in the catch-up cohort (*4*).

Previously, we reported that sustained high uptake of HPV vaccination was associated with reduced prevalence of HPV types 16 and 18 and evidence of cross-protection against nonvaccine types HPV 31, 33, and 45 among women who had undergone their first cervical screening test for precancerous disease from 2009 through 2012 (*5*). These data reconcile with studies undertaken in other settings. In England, an ecologic study showed that 19.1% and 6.5% of vaginal swab samples were positive for HPV 16 and 18 in the pre- and postvaccination periods, respectively (*6*). Markowitz et al. also demonstrated that despite low vaccine coverage, HPV 16 and 18 prevalence among girls who had received the quadrivalent vaccine in the routine and catch-up programs was reduced by 56% (*7*). Evidence is also emerging with regard to the effectiveness of HPV vaccination for reducing the incidence and prevalence of low- and high-grade precancerous cervical lesions (*8*–*11*). In Australia, recent studies designed to assess the extent of herd immunity to vaccine-type HPV have shown evidence for potential development of herd immunity in the nonvaccinated population (*12*,*13*). However, few studies of the extent of herd immunity have been published, particularly studies in which vaccination status can be directly linked to viral outcomes.

The ability to directly link large datasets (including cervical screening, vaccination, and disease registers) in Scotland through a unique personal identifier, the Community Health Index (CHI), enables us to comprehensively assess the effects of vaccination, including the extent of potential herd immunity. By including samples from women undergoing their first cervical smear testing in 2013, we further assessed the effects of HPV vaccination among young women in Scotland by comparing prevalence of HPV 16 and 18; HPV 31, 33, and 45; and other high-risk HPV types among women who were fully vaccinated as part of the catch-up cohort with prevalence among nonvaccinated women in the same birth cohorts. Additionally, we investigated whether the prevalence of any nonvaccine HPV types was greater among vaccinated women. Using these updated data, we determined whether high uptake of the vaccine protects nonvaccinated women by assessing the trend, over birth cohort, for the proportion of nonvaccinated women with positive results for each HPV outcome.

## Materials and Methods

### Surveillance Program and Sample Population

The Scottish Cervical Screening Programme is an organized, national, call–recall program that invites women 20–60 years of age to visit their general practitioner for a cervical smear test (*14*). The program is facilitated through the electronic Scottish Cytology Call–Recall System, which records which women are eligible for screening and contains information about cytology, histology, vaccination status, recall, and management.

During 2009–2013, the National Health Service cytopathology laboratories that serve the Screening Programme collected ≈1,000 liquid-based cytology samples per year from women 20–21 years of age who were undergoing their first cervical smear testing. All samples collected during 2009–2013 were subjected to HPV genotyping, and the results from the 2009–2010 samples constituted a prevaccination baseline. The sampling methods used in this study are described elsewhere (*5*).

### Data and Linkage

Liquid-based cytology samples collected by the cytology laboratories were labeled with an anonymous study identification number and underwent HPV genotyping at the Scottish HPV Reference Laboratory. The study identification numbers and the CHI number were sent to the Information Services Division of the Scottish National Health Service, where CHI numbers were used to link data from the Scottish Cytology Call–Recall System, the Scottish Immunisation Call–Recall System, and the Child Health Schools Program-System. The postal code of the patient’s residence was used to rank the geographic data zone for each sample according to the Scottish Index of Multiple Deprivation (1 = most deprived and 5 = least deprived; http://www.gov.scot/Topics/Statistics/SIMD/BackgroundMethodology). 

### HPV Testing

A detailed account of the testing procedures has been described elsewhere (*5*). In brief, HPV genotyping was performed by using the Multimetrix HPV Genotyping Kit (Diamex, Heidelberg, Germany), which can detect 24 HPV types, including all established high-risk carcinogenic types (HPV 16, 18, 31, 33, 35, 39, 45, 51, 52, 56, 58, and 59); probable carcinogenic types (HPV 68); and some possibly carcinogenic types (HPV 26, 53, 66, 70, 73, and 82), according to the latest International Agency for Research on Cancer groupings (*15*). This assay can also detect 5 low-risk HPV types (HPV 6, 11, 42, 43, and 44). International Agency for Research on Cancer guidelines also include HPV 67 as possibly carcinogenic, but this type is currently undetectable by use of the Multimetrix HPV kit (*16*).

### Statistical Analyses

Power calculations for the liquid-based cytology samples are described elsewhere (*5*). The prevalence of each detectable HPV type, along with 95% CIs, was calculated. A z-test of 2 proportions was used to assess differences in HPV type–specific prevalence among women who received all 3 doses of the vaccine and those who received none. The Bonferroni correction (significance level α = 0.05/22) was used because of the multiple statistical testing conducted for the 22 nonvaccine HPV types detected by the assay. Significance was assessed at α = 0.05 for HPV types 16 and 18. Association between the number of doses of vaccine received and HPV outcome was measured by using logistic regression adjusted for deprivation score, birth cohort year, and age at vaccination. A linear trend test was used to assess evidence for a linear change in positivity over the range of the previously mentioned variables. HPV outcomes were positivity for HPV types 16 or 18; HPV 31, 33 or 45; other nonvaccine high-risk types (HPV 35, 39, 51, 52, 56, 58, 59, and 68) in the carcinogenic and probably carcinogenic categories; or any HPV type detected by the Multimetrix HPV assay. Potential herd immunity was evaluated by using logistic regression and testing for a linear trend over time in the prevalence of HPV 16 and 18, the cross-protective types, other nonvaccine high-risk types, and any HPV among women who were not vaccinated during 2009–2013.

## Results

### Sample Characteristics

We analyzed 5,765 liquid-based cytology samples from women 20–21 years of age who underwent their first cervical smear testing during 2009–2013. The number of samples received each year was distributed evenly between the quintiles of the Scottish Index of Multiple Deprivation (≈20% samples/quintile) (Table 1). Overall, valid HPV test results were available for 5,715 samples, of which 57.1% (95% CI 55.8%–58.3%) were positive for any HPV type and 46.9% (95% CI 45.6%–48.2%) were positive for any high-risk HPV type (HPV 16, 18, 31, 33, 35, 39, 45, 51, 52, 56, 58, 59, 66, or 68). As expected, because of eligibility criteria, vaccination status differed greatly by collection year; 38% of women received 3 doses in 2011, 67% in 2012, and 72% in 2013. The samples received in 2009 and 2010 were from women who were not eligible for the catch-up campaign; therefore, 98% and 94% of the samples from these years, respectively, came from nonvaccinated women (Figure). 

**Table 1:** Yearly distribution of 5,765 liquid-based cytology samples collected from women 20–21 years of age undergoing their first cervical smear collection, Scotland, 2009–2013*

Year	Total	No. (%) samples										No. (%) samples with valid HPV results
		No. vaccine doses received					SIMD score					
		0	1	2	3		1	2	3	4	5	
2009	1,673	1,652 (98.74)	5 (0.30)	1 (0.06)	15 (0.90)		386 (23.07)	389 (23.25)	335 (20.02)	271 (16.20)	292 (17.45)	1,652 (98.74)
2010	1,074	1,012 (94.23)	7 (0.65)	7 (0.65)	48 (4.47)		260 (24.21)	208 (19.37)	219 (20.39)	193 (17.97)	194 (18.06)	1,053 (98.04)
2011	1,005	557 (55.42)	18 (1.79)	48 (4.78)	382 (38.01)		235 (23.38)	190 (18.91)	185 (18.41)	201 (20.00)	194 (19.30)	1,001 (99.60)
2012	997	245 (24.57)	26 (2.61)	52 (5.22)	674 (67.60)		216 (21.66)	201 (20.16)	172 (17.25)	191 (19.16)	217 (21.77)	993 (99.60)
2013	1,016	198 (19.49)	33 (3.25)	46 (4.53)	739 (72.74)		251 (24.70)	211 (20.77)	191 (18.80)	141 (13.88)	222 (21.85)	1,018 (100.00)

*HPV, human papillomavirus; SIMD, Scottish Index of Multiple Deprivation (1 = most deprived; 5 = least deprived).

**Figure:**
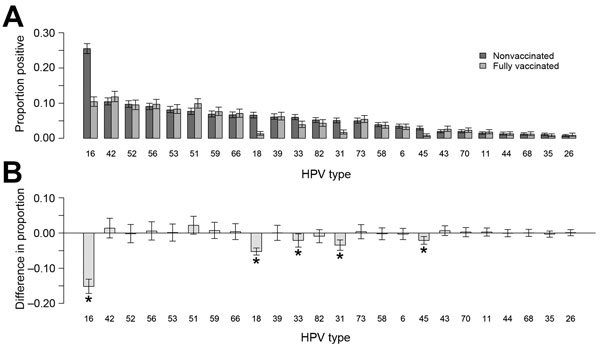
Analyses for 5,715 liquid-based cytology cervical samples from vaccinated and nonvaccinated women, for which valid human papillomavirus (HPV) testing results were available, Scotland, 2009–2013. A) Proportion and 95% CIs for samples with positive results for each HPV type. B) Difference in the proportion positive and associated 95% CIs for the difference between vaccinated and nonvaccinated women, by HPV type. Other than HPV types 16 and 18, the 95% CIs of the difference were corrected for multiple testing using by using the Bonferroni correction. *Significant change.

### Effect on Vaccine-Type Infections

We observed a statistically significant decrease in HPV 16 and 18 among vaccinated compared with nonvaccinated women (p<0.0001) (Figure). Positivity for HPV 16 and 18 in the samples was 11% (95% CI 9.7%–12.5%) among fully vaccinated women and 29.4% (95% CI 27.9%–30.9%) among nonvaccinated women (Table 2). Overall, annual prevalence of HPV 16 and 18 decreased over time; 10.1% (95% CI 8.4%–12.2%) of the samples collected in 2013 were positive for HPV 16 and 18, whereas 28.8% (95% CI 26.7%–31%) of the samples collected in 2009 were positive (*5*).

**Table 2:** Prevalence and unadjusted odds of positivity for HPV types 16 or 18 and cross-protective types stratified by year of sample collection, number of doses received, birth year, and age at vaccination, Scotland, 2009–2013 (N = 5,765)*

Variable	No.	HPV 16 or 18				Cross-protective HPV types†		
		No. pos	% Pos (95% CI)	Unadjusted OR (95% CI)		No. pos	% Pos (95% CI)	Unadjusted OR (95% CI)
Collection year								
2009	1,652	476	28.8 (26.7–31.0)	1 (reference)		215	13.0 (11.5–14.7)	1 (reference)
2010	1,053	333	31.6 (28.9–34.5)	1.14 (0.97–1.35)		143	13.6 (11.6–15.8)	1.05 (0.84–1.32)
2011	1,001	233	23.3 (20.7–26.0)	0.75 (0.63–0.90)		104	10.4 (8.7–12.4)	0.78 (0.60–0.99)
2012	993	169	17.0 (14.8–19.5)	0.51 (0.42–0.62)		83	8.4 (6.8–10.2)	0.61 (0.47–0.79)
2013	1,016	103	10.1 (8.40–12.2)	0.28 (0.22–0.35)		64	6.3 (5.0–8.0)	0.45 (0.33–0.60)
No. doses vaccine received								
0	3,619	1062	29.4 (27.9–30.9)	1 (reference)		468	12.9 (11.9–14.1)	1 (reference)
1	89	20	22.5 (15.0–32.2)	0.70 (0.41–1.13)		15	16.9 (10.5–26.0)	1.37 (0.75–2.33)
2	154	28	18.2 (12.9–25.0)	0.54 (0.35–0.80)		11	7.1 (4.0–12.3)	0.52 (0.26–0.92)
3	1,853	204	11.0 (9.70–12.5)	0.30 (0.25–0.35)		115	6.2 (5.2–7.4)	0.45 (0.36–0.55)
Birth year								
1988	844	251	29.7 (26.8–32.9)	1 (reference)		119	14.1 (11.9–16.6)	1 (reference)
1989	1,196	343	28.7 (26.2–31.3)	0.96 (0.79–1.17)		140	11.7 (10–13.7)	0.82 (0.63–1.06)
1990	1,204	349	29.0 (26.5–31.6)	0.97 (0.80–1.18)		155	12.9 (11.1–14.9)	0.91 (0.70–1.17)
1991	867	175	20.2 (17.6–23.0)	0.60 (0.48–0.74)		80	9.2 (7.5–11.3)	0.62 (0.46–0.83)
1992	1,261	169	13.4 (11.6–15.4)	0.36 (0.29–0.45)		90	7.1 (5.8–8.7)	0.47 (0.35–0.62)
1993	393	27	6.90 (4.8–9.8)	0.17 (0.11–0.26)		25	6.4 (4.3–9.2)	0.41 (0.26–0.63)
Age at vaccination, y‡								
15–16	970	75	7.7 (6.2–9.6)	1 (reference)		52	5.4 (4.1–7.0)	1 (reference)
17	631	79	12.5 (10.2–15.3)	1.70 (1.22–2.38)		47	7.4 (5.6–9.8)	1.42 (0.94–2.13)
18	391	65	16.6 (13.3–20.6)	2.38 (1.67–3.40)		30	7.7 (5.4–10.7)	1.47 (0.91–2.32)
>18	109	33	30.3 (22.4–39.5)	5.31 (3.28–8.48)		12	11 (6.4–18.3)	2.23 (1.1–4.18)

*HPV, human papillomavirus; OR, odds ratio; pos, positive. †HPV types 31, 33, or 45. ‡For those vaccinated.

Unadjusted analysis showed a significant linear trend for birth cohort and number of doses a woman received; prevalence of HPV 16 and 18 was lower among women from later birth cohorts and women who had received more doses (both p<0.0001) (Table 2). The linear trend remained significant for both variables in the adjusted analysis (both p<0.0001); however, the adjusted odds of positivity were tempered for birth cohort (Table 3). The adjusted odds ratio (OR) of being infected with HPV 16 or 18 decreased with every dose. For 1 dose, OR was 0.45 (95% CI 0.24–0.84); 2 doses, OR 0.39 (95% CI 0.23–0.67); and 3 doses, OR 0.27 (95% CI 0.19–0.37). Women from least deprived areas were significantly less likely to have positive results for HPV 16 and 18 than were those from more deprived areas (p = 0.0322). The linear trend for age at vaccination was significant; odds of infection with HPV 16 and 18 were greater for women vaccinated at older ages than for those vaccinated at 15–16 years of age (p<0.0001) (Table 3).

**Table 3:** Adjusted odds of positivity for HPV 16 or 18 and cross-protective HPV types by birth year, number of doses of vaccine received, SIMD score, and age at vaccination, Scotland, 2009–2013*

Variable	HPV type 16 or 18		Cross-protective HPV types†

Adjusted OR (95% CI)‡	Linear trend p value	Adjusted OR (95% CI)‡	Linear trend p value	
Birth year		0.0005			0.2413	
1988	1 (reference)			1 (reference)		
1989	0.96 (0.79–1.17)			0.82 (0.63–1.06)		
1990	1.02 (0.84–1.24)			0.97 (0.75–1.27)		
1991	0.97 (0.75–1.25)			0.86 (0.60–1.21)		
1992	0.84 (0.63–1.11)			0.77 (0.53–1.12)		
1993	0.43 (0.26–0.67)			0.72 (0.42–1.20)		
No. doses vaccine received		<0.0001			<0.0001	
0	1 (reference)			1 (reference)		
1	0.45 (0.24–0.84)			1.15 (0.54–2.33)		
2	0.39 (0.23–0.67)			0.46 (0.21–0.94)		
3	0.27 (0.19–0.37)			0.45 (0.29–0.68)		
SIMD quintile		0.0322			0.0028	
1 (most deprived)	1 (reference)			1 (reference)		
2	0.84 (0.70–1.02)			1.05 (0.82–1.33)		
3	0.85 (0.70–1.03)			0.93 (0.72–1.19)		
4	0.91 (0.75–1.11)			0.72 (0.54–0.94)		
5 (least deprived)	0.75 (0.62–0.92)			0.76 (0.58–0.99)		
Age at vaccination, y§		<0.0001			0.3736	
15–16	1 (reference)			1 (reference)		
17	1.33 (0.92–1.91)			1.26 (0.80–1.98)		
18	1.65 (1.07–2.53)			1.19 (0.67–2.08)		
>18	3.41 (1.98–5.82)			1.50 (0.68–3.12)		

*HPV, human papillomavirus; OR, odds ratio; SIMD, Scottish Index of Multiple Deprivation. †HPV types 31, 33, or 45. ‡Adjusted for birth year, SIMD score, and age at vaccination. §For those vaccinated.

### Evidence for Cross-Protection

Prevalence of HPV types 31, 33, and 45 decreased among vaccinated compared with nonvaccinated women (p<0.0001, p = 0.0012, and p<0.0001, respectively) (Figure). The positivity for cross-protective HPV types was 12.9% (95% CI 11.9%–14.1%) among nonvaccinated women and 6.2% (95% CI 5.2%–7.4%) among fully vaccinated women (Table 2). During 2009–2013, overall cross-protective type prevalence also declined, from 13% (95% CI 11.5%–14.7%) in 2009 to 6.3% (95% CI 5%–8%) in 2013 (*5*).

According to unadjusted analyses, the odds of being infected with cross-protective types decreased significantly according to birth cohort year (p = 0.0001), but adjusted analyses showed no such significant effect (p = 0.2413) because of confounding of the effect with vaccination status (Tables 2, 3). A strong significant linear trend was observed according to the number of doses received; the adjusted odds of positivity decreased with the number of doses received (1-dose OR 1.15 [95% CI 0.54–2.33] vs. 3-dose OR 0.45 [95% CI 0.29–0.68]; p<0.0001). Odds of positivity for cross-protective types were significantly reduced among women from the least deprived backgrounds (p = 0.0028); however, no significant difference was observed according to age at vaccination (p = 0.3736).

### Positivity for High-Risk HPV Types other than 16, 18, 31, 33, and 45

The overall prevalence of nonvaccine, non–cross-protective high-risk HPV types (HPV 35, 39, 51, 52, 56, 58, 59 and 68) significantly increased, from 29.1% (95% CI 26.9%–31.3%) in 2009 to 33.9% (95% CI 31.0%-36.8%) in 2013 (p = 0.0128) (*5*). Prevalence of nonvaccine, non–cross-protective high-risk HPV types did not differ significantly (p = 0.959) between nonvaccinated women (32.5% [95% CI 31%–34%]) and fully vaccinated women (32.9% ([95% CI 30.8%–35%]) (Table 4). Prevalence of HPV 51 was marginally and nonsignificantly increased among vaccinated women compared with nonvaccinated women (p = 0.0059).

**Table 4:** Prevalence and unadjusted odds of high-risk HPV excluding vaccine and cross-protective types and any HPV by year of sample collection, number of doses received, birth year, and age at vaccination, Scotland, 2009–2013 (N = 5,765)*

Variable	No.	High-risk HPV, excluding vaccine and cross-protective types†		Any HPV

No. pos	% Pos (95% CI)	Unadjusted OR (95% CI)	No. pos	% Pos (95% CI)	Unadjusted OR (95% CI)
Collection year								
2009	1,652	480	29.1 (26.9–31.3)	1 (reference)		959	58.1 (55.7–60.4)	1 (reference)
2010	1,053	364	34.6 (31.7–37.5)	1.29 (1.09–1.52)		618	58.7 (55.7–61.6)	1.01 (0.86–1.18)
2011	1,001	330	33.0 (30.1–35.9)	1.20 (1.01–1.42)		587	58.6 (55.6–61.7)	1.05 (0.89–1.23)
2012	993	352	35.5 (32.5–38.4)	1.34 (1.13–1.59)		580	58.4 (55.3–61.4)	1.04 (0.88–1.21)
2013	1,016	344	33.9 (31.0–36.8)	1.25 (1.06–1.48)		547	53.8 (50.8–56.8)	0.87 (0.74–1.02)
No. doses								
0	3,619	1,176	32.5 (31.0–34.0)	1 (reference)		2162	59.7 (58.1–61.3)	1 (reference)
1	89	32	36.0 (26.8–46.3)	1.17 (0.75–1.80)		55	61.8 (51.4–71.2)	1.12 (0.73–1.75)
2	154	53	34.4 (27.4–42.2)	1.09 (0.77–1.52)		91	59.0 (51.2–66.5)	1.00 (0.73–1.40)
3	1,853	609	32.9 (30.8–35.0)	1.02 (0.90–1.15)		983	53.1 (50.7–55.3)	0.78 (0.70–0.87)
Birth year								
1988	844	235	27.8 (24.9–31.0)	1 (reference)		477	56.5 (53.2–59.8)	1 (reference)
1989	1,196	371	31.0 (28.5–33.7)	1.18 (0.98–1.44)		700	58.5 (55.7–61.3)	1.09 (0.91–1.30)
1990	1,204	413	34.3 (31.7–37.0)	1.37 (1.13–1.66)		697	57.9 (55.1–60.6)	1.06 (0.89–1.26)
1991	867	287	33.1 (30.1–36.3)	1.28 (1.04–1.58)		515	59.4 (56.1–62.6)	1.13 (0.93–1.36)
1992	1,261	435	34.5 (31.9–37.2)	1.36 (1.12–1.64)		706	56.0 (53.2–58.7)	0.98 (0.82–1.17)
1993	393	129	32.8 (28.4–37.6)	1.25 (0.97–1.62)		196	49.9 (45.0–54.8)	0.77 (0.60–0.97)
Age at vaccination, y‡								
15–16	970	305	31.4 (28.6–34.4)	1 (reference)		491	50.6 (47.5–53.8)	1 (reference)
17	631	241	38.2 (34.5–42.0)	1.34 (1.09–1.66)		358	56.7 (52.8–60.5)	1.28 (1.05–1.57)
18	391	116	29.7 (25.4–34.4)	0.92 (0.71–1.19)		220	56.3 (51.3–61.1)	1.26 (0.99–1.59)
>18	109	32	29.4 (21.6–38.5)	0.93 (0.59–1.42)		60	55.0 (45.7–64.1)	1.19 (0.80–1.78)

*HPV, human papillomavirus; OR, odds ratio; pos, positive. †HPV 35, 39, 51, 52, 56, 58, 59, or 68. ‡For those vaccinated.

Odds of nonvaccine or cross-protective high-risk HPV type infection were significantly higher for women in later birth cohorts than for those in earlier birth cohorts (p = 0.0147) (Table 5). According to adjusted analysis, the odds of positivity for a nonvaccine, non–cross-protective, high-risk HPV type was 1.5 times higher for those born in 1992 and 1993 than for those born in 1988 (reference birth cohort). Although the unadjusted analysis shows some tempering of this effect, a linear trend was still present (p = 0.04) (Table 4). When adjusted for birth cohort, odds of infection were slightly reduced for women who received 3 doses of vaccine compared with women who received no vaccine, but this difference was not significant (p = 0.2953). No significant linear trend was found for nonvaccine, non–cross-protective, high-risk HPV type positivity according to deprivation status (p = 0.1378) or age at vaccination (p = 0.4541).

**Table 5:** Adjusted odds of positivity for high-risk HPV excluding vaccine and cross-protective types and any HPV type by birth year, number of doses received, SIMD score, and age at vaccination, Scotland, 2009–2013*

Variable	High-risk HPV types, excluding vaccine and cross-protective types†			Any HPV	
	Adjusted OR (95% CI)‡	Linear trend p value		Adjusted OR (95% CI)‡	Linear trend p value
Birth year		0.0147			0.1158
1988	1 (reference)			1 (reference)	
1989	1.18 (0.97–1.44)			1.08 (0.91–1.29)	
1990	1.41 (1.16–1.72)			1.10 (0.92–1.32)	
1991	1.32 (1.04–1.69)			1.36 (1.08–1.71)	
1992	1.49 (1.16–1.91)			1.39 (1.10–1.77)	
1993	1.46 (1.06–2.00)			1.13 (0.84–1.53)	
No. doses vaccine received		0.2953			0.004
0	1 (reference)			1 (reference)	
1	0.89 (0.53–1.48)			0.78 (0.48–1.30)	
2	0.84 (0.56–1.27)			0.72 (0.48–1.07)	
3	0.80 (0.63–1.02)			0.60 (0.48–0.76)	
SIMD quintile		0.1378			0.0002
1 (most deprived)	1 (reference)			1 (reference)	
2	0.99 (0.84–1.17)			0.93 (0.80–1.10)	
3	0.99 (0.84–1.18)			0.83 (0.70–0.97)	
4	0.98 (0.82–1.17)			0.87 (0.74–1.03)	
5 (least deprived)	0.87 (0.73–1.03)			0.73 (0.62–0.86)	
Age at vaccination, y§		0.4541			0.331
15–16	1 (reference)			1 (reference)	
17	1.40 (1.10–1.77)			1.2 (0.95–1.51)	
18	0.98 (0.71–1.35)			1.24 (0.92–1.68)	
>18	0.98 (0.60–1.57)			1.25 (0.80–1.96)	

*HPV, human papillomavirus; OR, odds ratio; SIMD, Scottish Index of Multiple Deprivation. †HPV 35, 39, 51, 52, 56, 58, 59, or 68. ‡Adjusted for birth cohort year, SIMD score, and age at vaccination.  §For those vaccinated.

### Overall Positivity for any HPV Type

Prevalence of all 24 HPV types detected by the assay remained unchanged from 2009 to 2012 (58.1% [95% CI 55.7%–60.4%] in 2009 and 58.4% [95% CI 55.3%–61.4%] in 2012) but decreased to 53.8% (95% CI 50.8%–56.9%) in 2013 (Table 4) (*5*). Overall HPV positivity was 53.1% (95% CI 50.8%–55.3%) among fully vaccinated women and higher (59.7% [95% CI 58.1%–61.3%]) among nonvaccinated women.

According to unadjusted analyses, overall HPV positivity showed a significant linear trend by birth cohort year; HPV infection was more likely among women in later birth cohorts than among than those born in 1988 (p = 0.03171). According to adjusted analyses, however, this trend was not significant (p = 0.115) (Tables 4, 5). HPV infection was significantly less likely among women who had received 3 doses of vaccine than among those who had received no doses (p<0.004) and was also less likely among women from the least deprived backgrounds than among those from the most deprived backgrounds (linear trend test p = 0.0002). Furthermore, overall HPV positivity did not differ significantly between women vaccinated at different ages (p = 0.331).

### Prevalence of HPV among Nonvaccinated Women (Herd Immunity)

Prevalence of HPV 16 and 18 among nonvaccinated women remained relatively stable at ≈30% during 2009–2012 but decreased to 21.2% in 2013 (Table 6). During 2010–2013, prevalence of HPV types 31, 33, or 45 declined gradually, from 13.7% in 2010 to 9.6% in 2013. In 2013, the odds of infection with HPV types 16 and 18 was reduced among nonvaccinated women (OR 0.67 [95% CI 0.47–0.96]) compared with the baseline odds in 2009, and testing for trend over all years showed a marginal decrease over time (p = 0.054) (Table 6). Odds of infection with HPV types 31, 33, or 45 were reduced in 2012 and 2013 compared with 2009, but these odds were not significant, and no significant linear trend was observed (p = 0.104). The odds of infection with nonvaccine, non–cross-protective, high-risk HPV types were significantly higher among nonvaccinated women in 2010, 2011, and 2012 (OR 1.26 [95% CI 1.07–1.5], OR 1.5 [95% 1.22–1.83], and OR 1.44 [95% CI 1.09–1.91], respectively) than in 2009 (Table 7). Odds of infection with any HPV were increased in 2011 and 2012 (OR 1.3 [95% CI 1.07–1.59] and OR 1.56 [95% CI 1.18–2.09], respectively) over odds in 2009, but this linear trend was not significant (p = 0.0576) (Table 7).

**Table 6:** Prevalence and odds of infection with HPV types 16 or 18 and for HPV cross-protective types among nonvaccinated women, by study year, Scotland, 2009–2013*

Study year	No. women	HPV 16 or 18				Cross-protective HPV types†		
		No. pos	% Pos (95% CI)	OR (95% CI)		No. pos	% Pos (95% CI)	OR (95% CI)
2009	1,652	468	28.3 (26.2–30.6)	1 (reference)		211	12.8 (11.2–14.5)	1 (reference)
2010	1,012	310	30.6 (27.9–33.5)	1.13 (0.95–1.34)		139	13.7 (11.8–16.0)	1.10 (0.87–1.38)
2011	557	164	29.4 (25.8–33.4)	1.05 (0.85–1.29)		71	12.7 (10.2–15.8)	0.99 (0.74–1.32)
2012	245	78	31.8 (26.3–37.9)	1.18 (0.88–1.57)		28	11.4 (8.0–16.0)	0.88 (0.58–1.33)
2013	198	42	21.2 (16.1–27.4)	0.67 (0.47–0.96)		19	9.6 (6.2–14.5)	0.71 (0.44–1.17)

*HPV, human papillomavirus; OR, odds ratio; pos, positive.  †HPV 31, 33, or 45.

**Table 7:** Prevalence and odds of infection with high-risk HPV excluding vaccine and cross-protective types and for any HPV among nonvaccinated women, by study year, Scotland, 2009–2013*

Study year	No. women	High-risk HPV excluding vaccine and cross-protective types†				Any HPV		
		No. pos	% Pos (95% CI)	OR (95% CI)		No. pos	% Pos (95% CI)	OR (95% CI)
2009	1.652	473	28.6 (26.5–30.9)	1		946	57.3 (54.9–59.6)	1
2010	1,012	338	33.4 (30.6–36.4)	1.26 (1.07–1.5)		578	57.1 (54.0–60.1)	0.99 (0.85–1.16)
2011	557	210	37.7 (33.8–41.8)	1.50 (1.22–1.83)		354	59.5 (59.5–67.4)	1.30 (1.07–1.59)
2012	245	90	36.7 (30.9–42.9)	1.44 (1.09–1.91)		166	61.7 (61.7–73.3)	1.56 (1.18–2.09)
2013	198	65	32.8 (26.7–39.6)	1.20 (0.87–1.64)		118	52.6 (53.6–66.2)	1.10 (0.82–1.49)

*HPV, human papillomavirus; OR, odds ratio; pos, positive.  †HPV 35, 39, 51, 52, 56, 58, 59, or 68.

## Discussion

Decline of HPV prevalence in Scotland has been reported (*5*). We show a further decline in the prevalence of HPV types 16 and 18 among women in Scotland, associated with high rates of vaccination with the bivalent HPV vaccine. There is evidence that each dose administered conferred protection against HPV type 16 or 18 and that the odds of infection were significantly reduced after 2 and 3 doses. Our findings are comparable with those of a nested analysis of a randomized controlled trial, which reported efficacy of 1, 2, and 3 doses of bivalent HPV vaccine against HPV 16 and 18 infection (*17*). However, because of the small number of women in our study who had received 2 doses and because our study was powered to detect an effect of 3 doses, the findings with regard to effectiveness of <3 doses should be considered preliminary and may be confounded by other factors. Our evidence of the vaccine conferring cross-protection against HPV types 31, 33, and 45 is also consistent with findings of a previous study (*18*). Of note, published results of a recent study in England (*6*) do not report similar reductions in cross-protective types; however, the authors of that article concluded that further analysis of samples from women in birth cohorts with high vaccine coverage was needed for full evaluation of the effects of vaccination on prevalence of nonvaccine HPV types. 

We observed a reduction in the prevalence of HPV types 16 and 18 among nonvaccinated women undergoing their first cervical smear testing in 2013. This finding is encouraging evidence of herd immunity and corroborates the findings of 2 studies from Australia: a sexual health clinic–based national surveillance study that observed a decline in genital warts among heterosexual men and a cross-sectional study that showed a decrease in HPV prevalence among nonvaccinated women (*12*,*13*). These data and our data are welcome because protecting the nonvaccinated population from infection with HPV 16 or 18 depends on development of herd immunity. However, the limited number of nonvaccinated women, particularly in later years since the introduction of the program, makes interpretation of these results somewhat challenging, as reflected in the CIs. In addition, although in 2009, nonvaccinated women were not vaccinated because of ineligibility, in 2013, nonvaccinated women may not have been vaccinated for other reasons (religious, cultural, or societal), which may influence their likelihood of being infected by HPV types 16 or 18 relative to women in the 2009 cohort. However, the increased odds of infection with nonvaccine, non–cross-protective, high-risk HPV types and any HPV type among nonvaccinated women in years after 2009 suggests that these reasons are probably not a major confounding factor. Data from future cohorts will show whether the reduction of infection among nonvaccinated women is sustained.

It has been postulated that the reduction of infection with HPV types 16 and 18 and cross-protective types could leave a vacant niche, leading to increased infections with less oncogenic, nonvaccine HPV types among vaccinated women (*19*,*20*). We found that odds of infection with nonvaccine, non–cross-protective, high-risk HPV types were higher among women in later birth cohorts than among those in earlier birth cohorts. Furthermore, we observed that the most common high-risk type infecting fully vaccinated women in later cohorts was HPV 51, replacing HPV 16 as the most prevalent type but at lower rates. However, because of the increased overall prevalence between 2009 and 2013, comparison of nonvaccine high-risk HPV type prevalence between nonvaccinated and fully vaccinated women will be confounded. It is feasible that rather than truly replacing vaccine types, the other high-risk types are simply being unmasked because of less competition for the resources within molecular amplification assays (*21*). Consequently, we found no strong evidence for type replacement occurring in Scotland; these results are consistent with data from Australia (*22*). Continued follow-up is needed for evaluation of the potential for type replacement after high uptake of the bivalent HPV vaccine. Our ongoing analysis of HPV prevalence among women with histologically confirmed cervical lesions and linkage to colposcopy data to assess cervical disease in the female population of Scotland will aid in addressing the issue of clinically relevant type replacement (*7*).

The main strength of this study is that vaccination and screening data can be linked at the individual level. Coupled with the high levels of vaccine uptake and age of screening in Scotland, this linkage enables us to directly evaluate the effects of HPV vaccination on HPV prevalence. 

Nevertheless, our study has some limitations. Data from a screened population are not representative of the whole population and possibly underestimate the true prevalence of HPV infection. However, the prevalence of HPV among 20-year-old women who did not undergo a cervical screening test but were invited to send a self-collected urine sample or vaginal swab sample did not differ significantly from prevalence among those who had undergone cervical screening testing in a previous study (*23*). We were also unable to collect sexual history data and so could not determine an overall change in sexual practices among the population, which might confound our results. However, our observed increase in prevalence of nonvaccine, non–cross-protective, high-risk HPV types among vaccinated and nonvaccinated women and the relative stability of the prevalence of HPV overall suggests either an increase or stabilization of sexual behavior between 2009 and 2013. Also, the results of the third National Surveys of Sexual Attitudes and Lifestyles study, conducted in 2013, showed that the number of women’s lifetime sex partners, a known risk factor for HPV infection, has increased since 2000, and it is therefore more likely that the effect of the vaccine has been underestimated in our study (*24*). In addition, the incidence of genital herpes and gonorrhea in Scotland increased from 2005 to 2014, suggesting that sexual activity has increased over time (*25*). However, women in later birth cohorts within the catch-up campaign are more likely to have received the vaccine within the school-based program than were those in earlier birth cohorts, who are more likely to have received the vaccine out of school (in general practice). Previous studies have shown that those who leave school are more likely to be from high-deprivation backgrounds and are consequently more likely to be infected with HPV (*4*,*26*). Therefore, the effect of the HPV vaccine may be confounded by differences between those who leave school and those who stay in school.

Our data preliminarily suggest the presence of herd immunity in the nonvaccinated female population of Scotland. However, we could not assess whether herd immunity is conferring protection to the male population, who are not routinely sampled as part of the surveillance program. We plan to use genital wart consultation data from men to act as a proxy for detecting herd immunity in the male population (*13*). In the meantime, the first girls who received the vaccine as part of the routine vaccination program will be eligible for cervical screening toward the end of 2015. Those data will enable us to demonstrate the effect of equitable >90% vaccine uptake on HPV prevalence and cervical disease among young, presumed HPV naive, women in Scotland.
